# COVID-19 outcomes in patients with inflammatory rheumatic diseases: associations with disease subtypes, treatments, and vaccination status

**DOI:** 10.3389/fpubh.2026.1907698

**Published:** 2026-09-22

**Authors:** Feyza Ak, Zeynep Kaya

**Affiliations:** 1 Division of Rheumatology, Department of Internal Medicine, Istanbul Bezmi Alem University Hospital, Istanbul, Türkiye; 2 Division of Rheumatology, Department of Internal Medicine, Istanbul Training and Research Hospital, Istanbul, Türkiye

**Keywords:** BNT162b2, CoronaVac, COVID-19, inflammatory rheumatic diseases, vaccination

## Abstract

**Background:**

Patients with inflammatory rheumatic diseases (IRDs) may be at increased risk for adverse COVID-19 outcomes because of immune dysregulation, comorbidities, and immunomodulatory treatments. However, the effects of specific rheumatic disease subsets, treatment patterns, and vaccine types remain incompletely understood.

**Objective:**

To evaluate clinical characteristics and factors associated with COVID-19 infection and hospitalization among patients with IRDs.

**Methods:**

This retrospective observational cohort study included 1,024 adult patients with IRDs followed between March 2020 and January 2023. Demographic characteristics, rheumatic disease subtypes, treatments, vaccination status, COVID-19 infection, hospitalization, radiologic findings, and recovery duration were analyzed.

**Results:**

Among all patients, 347 (33.9%) developed COVID-19 infection. Patients with COVID-19 were younger than those without COVID-19 (49.1 ± 13.4 vs. 51.5 ± 14.5 years, *p* = 0.007). Vaccination before infection was significantly more frequent in the COVID-19-negative group (92.3% vs. 23.9%, *p* < 0.001). Spondyloarthritis frequency was higher among COVID-19-positive patients (25.9% vs. 20.1%, *p* = 0.030), whereas methotrexate use was lower (14.4% vs. 22.6%, *p* = 0.002). Among COVID-19-positive patients, 40 (11.5%) required hospitalization. Hospitalized patients were older, had more frequent lung disease, longer rheumatic disease duration, increased radiologic involvement, and prolonged recovery duration (all *p* < 0.05). In univariate analyses, FMF, colchicine use, and BNT162b2 vaccination were associated with lower hospitalization rates; however, these associations were not retained after multivariable adjustment.

**Conclusion:**

Older age, pulmonary comorbidity, longer disease duration, and radiologic involvement were associated with COVID-19-related hospitalization in IRDs. In contrast, FMF, colchicine use, and BNT162b2 vaccination were associated with lower hospitalization rates.

## 1 Introduction

COVID-19 has created major challenges in the management of patients with inflammatory rheumatic diseases (IRDs) because of immune system dysregulation, chronic inflammation, accompanying comorbidities, and the frequent use of immunomodulatory therapies. During the early stages of the pandemic, patients with rheumatic diseases were considered potentially vulnerable to severe acute respiratory syndrome coronavirus-2 (SARS-CoV-2) infection and severe COVID-19 outcomes (1). Subsequent studies demonstrated that the clinical course of COVID-19 in IRDs is heterogeneous and influenced by several factors including age, comorbidities, disease activity, and antirheumatic treatments (2, 3).

Large international registries and multicenter cohort studies identified advanced age, cardiovascular disease, chronic lung disease, diabetes mellitus, and glucocorticoid exposure as important predictors of hospitalization and mortality among patients with rheumatic diseases (4, 5). In contrast, biologic agents and several conventional disease-modifying antirheumatic drugs (DMARDs) were not consistently associated with worse COVID-19 outcomes, suggesting that baseline patient characteristics and systemic inflammatory burden may play a greater role than immunosuppressive therapy alone (6, 7). Nevertheless, the influence of specific rheumatic disease subsets on COVID-19 susceptibility and severity remains incompletely clarified.

Rheumatoid arthritis (RA) and spondyloarthritis (SpA) are among the most commonly studied inflammatory rheumatic disorders in relation to COVID-19. Previous studies suggested that patients with SpA may have distinct infection patterns and hospitalization risks compared with RA or connective tissue diseases (CTDs) (8, 9). CTDs have also attracted attention because of their multisystem involvement and the frequent use of corticosteroids or immunosuppressive agents, which may contribute to severe infection outcomes (7). However, available data regarding comparative risks among different rheumatic disease categories remain inconsistent.

Familial Mediterranean fever (FMF), a prevalent autoinflammatory disease in Türkiye and neighboring regions, represents another clinically important subgroup. FMF is characterized by pyrin inflammasome activation and chronic innate immune dysregulation (10). Colchicine, the cornerstone treatment of FMF, gained considerable attention during the COVID-19 pandemic because of its anti-inflammatory and potential antiviral effects through inhibition of neutrophil chemotaxis and inflammasome-mediated cytokine release (11). Early clinical studies suggested that colchicine might reduce inflammatory complications and improve outcomes in COVID-19, although subsequent reports produced conflicting results (12, 13). Therefore, the role of colchicine in preventing severe COVID-19 outcomes in patients with rheumatic diseases remains uncertain.

Vaccination against SARS-CoV-2 has significantly reduced COVID-19-related morbidity and mortality worldwide. However, concerns persist regarding vaccine immunogenicity and breakthrough infections among patients receiving immunosuppressive therapies (13). Previous studies demonstrated that mRNA vaccines may induce stronger humoral immune responses compared with inactivated vaccines in patients with autoimmune inflammatory diseases (14). In Türkiye, both the inactivated CoronaVac (Sinovac) vaccine and the mRNA BNT162b2 (Pfizer-BioNTech) vaccine were extensively used during the pandemic, allowing comparative evaluation of vaccine-associated outcomes in real-life rheumatic disease cohorts. Despite increasing evidence regarding vaccine efficacy in autoimmune diseases, real-world comparative data regarding different vaccine types in IRDs remain limited.

The Turkish Society for Rheumatology COVID-19 Registry provided important nationwide data on the course of COVID-19 among patients with IRDs and identified older age, comorbidities, and glucocorticoid use as major determinants of adverse outcomes (15). However, additional real-life studies evaluating disease-specific characteristics, treatment patterns, vaccination status, and hospitalization-associated factors are still needed, particularly in populations with substantial numbers of FMF and SpA patients.

Therefore, in this study, we aimed to evaluate the demographic and clinical characteristics of patients with IRDs according to COVID-19 infection status and to investigate factors associated with COVID-19-related hospitalization, with particular emphasis on rheumatic disease subtypes, antirheumatic treatments, colchicine use, and vaccination patterns.

## 2 Materials and methods

This retrospective observational cohort study included consecutive adult patients with IRDs who were followed at the rheumatology outpatient clinic between March 2020 and January 2023. Patients aged ≥18 years with a confirmed diagnosis of inflammatory rheumatic disease according to established international classification criteria were enrolled. The study population included patients with RA, SpA, FMF, CTDs, Behçet disease, vasculitis, and other IRDs. The CTD group included patients with systemic lupus erythematosus, systemic sclerosis, idiopathic inflammatory myopathies, Sjögren’s syndrome, mixed connective tissue disease, and undifferentiated connective tissue disease.

Patients with incomplete clinical records, uncertain COVID-19 diagnosis, or insufficient follow-up data were excluded from the study.

Demographic, clinical and treatment-related data were retrospectively extracted from institutional electronic medical records and outpatient follow-up records. Recorded variables included age, sex, body mass index (BMI), smoking status, rheumatic disease subtype, disease duration, comorbid diseases, medications, vaccination history, COVID-19 infection status, hospitalization, intensive care unit (ICU) admission, radiologic findings, and recovery duration.

Comorbid conditions included diabetes mellitus (DM), hypertension (HT), coronary artery disease (CAD), thyroid disease, asthma/chronic obstructive pulmonary disease (COPD), and other chronic disorders.

Medication history at the time of COVID-19 infection or last clinical evaluation included corticosteroids, methotrexate (MTX), leflunomide (LEF), sulfasalazine (SLZ), hydroxychloroquine (HCQ), colchicine, biologic therapies, and nonsteroidal anti-inflammatory drugs (NSAIDs). Prednisone doses were recorded as mg/day.

Vaccination history prior to COVID-19 infection was also recorded. Patients were categorized according to vaccine type as BNT162b2 (Pfizer-BioNTech), CoronaVac (Sinovac), or combined vaccination.

COVID-19 diagnosis was established according to the Turkish Ministry of Health COVID-19 guidelines and was based on positive SARS-CoV-2 polymerase chain reaction (PCR) testing, compatible thoracic imaging findings when PCR testing was unavailable or inconclusive, or clinically confirmed infection according to the prevailing national recommendations (16). Patients were divided into COVID-19-positive and COVID-19-negative groups.

Patients were classified as COVID-19-negative if they had no documented diagnosis of COVID-19 throughout the follow-up period. Systematic screening for SARS-CoV-2 infection was not performed. Diagnostic testing was undertaken according to national pandemic guidelines, primarily in symptomatic individuals or those with suspected exposure.

Radiologic involvement was defined as pulmonary findings compatible with COVID-19 pneumonia on thoracic imaging. Recovery duration was defined as the time from symptom onset to complete clinical recovery.

The primary outcome of the study was to compare demographic and clinical characteristics between patients with and without COVID-19 infection. Secondary outcomes included identification of factors associated with COVID-19-related hospitalization and severe disease course.

Hospitalization decisions were based on the recommendations of the Turkish Ministry of Health COVID-19 guidelines, considering clinical severity, oxygen requirement, radiological findings, comorbidities, and the treating physician’s clinical judgment (17). As all patients were managed at the same institution, hospitalization decisions followed a consistent institutional approach throughout the study period.

Hospitalization due to COVID-19, ICU admission, duration of hospitalization, ICU stay duration, and pulmonary radiologic involvement were evaluated as indicators of disease severity.

### 2.1 Statistical analysis

Statistical analyses were performed using IBM SPSS Statistics for Windows, version 28.0 (IBM Corp., Armonk, NY, United States). Descriptive statistics were expressed as mean, standard deviation (SD), median, minimum–maximum values, frequency, and percentage, as appropriate.

The distribution of variables was evaluated using the Kolmogorov–Smirnov test. Independent quantitative variables were compared using the independent samples t-test or Mann–Whitney U test according to data distribution. Qualitative independent variables were analyzed using the chi-square test, and Fisher’s exact test was used when chi-square test assumptions were not met. Variables with a *p* value <0.05 in the univariate analyses were entered into multivariable logistic regression models using the Forward Likelihood Ratio (Forward LR) method to identify independent factors associated with COVID-19 infection and COVID-19-related hospitalization. Odds ratios (ORs) and 95% confidence intervals (95% CIs) were calculated for all regression models.

A two-sided *p* value <0.05 was considered statistically significant.

### 2.2 Ethical approval

The study protocol was approved by the Fatih Sultan Mehmet Training and Research Hospital ethics committee (Approval No: 2022/23, Date: 08/12/2022) in accordance with the principles of the Declaration of Helsinki. Written informed consent was obtained from all participants in accordance with institutional requirements and the Declaration of Helsinki.

## 3 Results

A total of 1,024 patients with inflammatory rheumatic diseases were included in the study. The mean age was 50.7 ± 14.2 years, and 71.8% of the patients were female. Rheumatoid arthritis (35.4%) was the most common diagnosis, followed by spondyloarthritis (22.1%) and connective tissue diseases (15.9%) (Table 1).

**Table 1:** Baseline demographic and clinical characteristics.

Variable	Subgroup	Min–Max	Median	Mean ± SD/n (%)
Age (years)		18.0–89.0	52.0	50.7 ± 14.2
Sex	Female			735 (71.8%)
	Male			289 (28.2%)
BMI (kg/m^2^)		15.4–48.4	28.3	28.8 ± 5.7
Smoking	Current smoker			228 (22.3%)
Comorbidities	DM			47 (4.6%)
	HT			81 (7.9%)
	Thyroid disease			43 (4.2%)
	CAD			35 (3.4%)
	Asthma/COPD			31 (3.0%)
	Other			32 (3.1%)
Disease duration (years)		0.0–45.0	5.0	7.4 ± 8.3
Rheumatic disease	RA			363 (35.4%)
	SpA			226 (22.1%)
	FMF			109 (10.6%)
	CTD			163 (15.9%)
	Behçet disease			75 (7.3%)
	Vasculitis			10 (1.0%)
	Other			78 (7.6%)
Medications	Prednisone			234 (22.9%)
	NSAIDs			180 (17.6%)
	MTX			203 (19.8%)
	LEF			105 (10.3%)
	SLZ			181 (17.7%)
	HCQ			234 (22.9%)
	Colchicine			200 (19.5%)
	Other			47 (4.6%)
	Biologic therapy			63 (6.2%)
Vaccination status before COVID-19	Vaccinated			708 (69.1%)
	BNT162b2 vaccine			310 (43.8%)
	CoronaVac			153 (21.6%)
	Combined vaccination			245 (34.6%)
Hospitalization				40 (3.9%)
Hospitalization duration (days)		2.0–32.0	10.0	10.6 ± 6.8
ICU admission				20 (2.0%)
ICU stay duration (days)		2.0–30.0	7.0	12.4 ± 10.3
Recovery duration (days)		2.0–100.0	14.0	17.7 ± 15.6

BMI, body mass index; DM, diabetes mellitus; HT, hypertension; CAD, coronary artery disease; COPD, chronic obstructive pulmonary disease; RA, rheumatoid arthritis; SpA, spondyloarthritis; FMF, familial Mediterranean fever; CTD, connective tissue disease; NSAIDs, nonsteroidal anti-inflammatory drugs; MTX, methotrexate; LEF, leflunomide; SLZ, sulfasalazine; HCQ, hydroxychloroquine; ICU, intensive care unit.

Among all patients, 347 (33.9%) developed COVID-19 infection. Patients with COVID-19 were younger than COVID-19-negative patients (49.1 ± 13.4 vs. 51.5 ± 14.5 years, *p* = 0.007). Vaccination before COVID-19 infection was significantly more common in the COVID-19-negative group (92.3% vs. 23.9%, *p* < 0.001). BNT162b2 and combined vaccination were more frequent among COVID-19-negative patients, whereas CoronaVac vaccination was more common among COVID-19-positive patients (all *p* < 0.001). In addition, spondyloarthritis frequency was significantly higher in COVID-19-positive patients (25.9% vs. 20.1%, *p* = 0.030), while methotrexate use was significantly lower (14.4% vs. 22.6%, *p* = 0.002) (Table 2).

**Table 2:** Comparison of patients with and without COVID-19.

Variable	COVID-19-negative (*n* = 677)	COVID-19-positive (*n* = 347)	*p* value
Age, years	51.5 ± 14.5	49.1 ± 13.4	0.007
Female sex	487 (71.9%)	248 (71.5%)	0.876
Vaccinated before COVID-19	625 (92.3%)	83 (23.9%)	<0.001
Combined vaccination	229 (36.6%)	16 (19.3%)	<0.001
BNT162b2 vaccine	287 (45.9%)	23 (27.7%)	<0.001
CoronaVac	109 (17.4%)	44 (53.0%)	<0.001
RA	248 (36.6%)	115 (33.1%)	0.269
SpA	136 (20.1%)	90 (25.9%)	0.030
CTD	118 (17.4%)	45 (13.0%)	0.070
FMF	65 (9.6%)	44 (12.7%)	0.131
MTX use	153 (22.6%)	50 (14.4%)	0.002
LEF use	77 (11.4%)	28 (8.1%)	0.099
SLZ use	112 (16.5%)	69 (19.9%)	0.185
HCQ use	161 (23.8%)	73 (21.1%)	0.322
Prednisone dose, mg/day	7.0 ± 8.8	5.3 ± 3.5	0.045
Biologics use	43 (6.4%)	20 (5.8%)	0.711

RA, rheumatoid arthritis; SpA, spondyloarthritis; CTD, connective tissue disease; FMF, familial Mediterranean fever; MTX, methotrexate; LEF, leflunomide; SLZ, sulfasalazine; HCQ, hydroxychloroquine. Continuous variables were compared using the independent samples t-test or Mann–Whitney U test according to data distribution. Categorical variables were compared using the chi-square test or Fisher’s exact test, as appropriate.

Among COVID-19-positive patients, 40 (11.5%) required hospitalization. Hospitalized patients were older (57.0 ± 12.4 vs. 48.1 ± 13.2 years, *p* < 0.001), had a higher frequency of lung disease (20.0% vs. 7.5%, *p* = 0.009), longer rheumatic disease duration (9.9 ± 9.8 vs. 7.1 ± 8.1 years, *p* = 0.040), and more frequent radiologic involvement (65.0% vs. 10.1%, *p* < 0.001). Recovery duration was also significantly longer in hospitalized patients (33.4 ± 25.2 vs. 15.1 ± 11.6 days, *p* < 0.001). FMF frequency (2.5% vs. 14.0%, *p* = 0.040), colchicine use (10.0% vs. 24.4%, *p* = 0.041), and BNT162b2 vaccination (0% vs. 31.9%, *p* = 0.030) were significantly lower among hospitalized patients (Table 3).

**Table 3:** Factors associated with COVID-19 hospitalization.

Variable	Non-hospitalized (*n* = 307)	Hospitalized (*n* = 40)	*p* value
Age, years	48.1 ± 13.2	57.0 ± 12.4	<0.001
Female	222 (72.3%)	26 (65.0%)	0.335
BMI	28.7 ± 5.7	29.8 ± 5.6	0.055
Smoking	68 (22.1%)	9 (22.5%)	0.960
Comorbid diseases	143 (46.6%)	23 (57.5%)	0.193
Lung disease	23 (7.5%)	8 (20.0%)	0.009
Rheumatic disease duration, years	7.1 ± 8.1	9.9 ± 9.8	0.040
RA	100 (32.6%)	15 (37.5%)	0.534
SpA	85 (27.7%)	13 (32.5%)	0.653
CTD	37 (12.1%)	5 (12.5%)	1.000
FMF	43 (14.0%)	1 (2.5%)	0.040
MTX use	41 (13.4%)	9 (22.5%)	0.121
LEF use	24 (7.8%)	4 (10.0%)	0.634
SLZ use	58 (18.9%)	11 (27.5%)	0.200
HCQ use	63 (20.5%)	10 (25.0%)	0.513
Colchicine use	75 (24.4%)	4 (10.0%)	0.041
Biologics use	18 (5.9%)	2 (5%)	0.826
CoronaVac	35 (48.6%)	9 (81.8%)	0.053
BNT162b2 vaccine	23 (31.9%)	0 (0%)	0.030
Combined vaccination	14 (19.4%)	2 (18.2%)	1.000
Radiologic findings	31 (10.1%)	26 (65.0%)	<0.001
Recovery duration, days	15.1 ± 11.6	33.4 ± 25.2	<0.001

BMI, body mass index; RA, rheumatoid arthritis; SpA, spondyloarthritis; CTD, connective tissue disease; FMF, familial Mediterranean fever; MTX, methotrexate; LEF, leflunomide; SLZ, sulfasalazine; HCQ, hydroxychloroquine. Continuous variables were compared using the independent samples t-test or Mann–Whitney U test according to data distribution. Categorical variables were compared using the chi-square test or Fisher’s exact test, as appropriate.

Univariate logistic regression analysis identified younger age, pre-COVID-19 vaccination status, BNT162b2 vaccination, CoronaVac vaccination, combined vaccination, spondyloarthritis, Sjögren syndrome, and methotrexate use as factors significantly associated with COVID-19 infection. In the multivariable logistic regression analysis, pre-COVID-19 vaccination status (OR 0.014, 95% CI 0.008–0.023, *p* < 0.001), CoronaVac vaccination (OR 6.373, 95% CI 3.692–11.00, *p* < 0.001), and combined vaccination (OR 0.195, 95% CI 0.111–0.343, *p* < 0.001) remained independently associated with COVID-19 infection (Table 4).

**Table 4:** Univariate and multivariate logistic regression analysis of factors associated with COVID-19 infection.

	Univariate			Multivariate		
	OR	95% CI	*p*	OR	95% CI	*p*
Age	0.988	0.979–0.997	***0.012***			
Pre-COVID-19 vaccination status	0.026	0.018–0.038	***0.000***	0.014	0.008–0.023	***0.000***
BNT162b2 vaccination	0.739	0.553–0.988	***0.041***			
CoronaVac vaccination	0.406	0.308–0.536	***0.000***	6.373	3.692–11.00	***0.000***
Combined vaccination	0.241	0.166–0.349	***0.000***	0.195	0.111–0.343	***0.000***
SpA	1.426	1.034–1.965	***0.030***			
Sjögren syndrome	0.478	0.256–0.891	***0.020***			
Prednisone	0.929	0.847–1.018	0.117			
Methotrexate use	0.577	0.406–0.818	***0.002***			

OR, odds ratio; CI, confidence interval; SpA, spondyloarthritis; MTX, methotrexate; BNT162b2, Pfizer–BioNTech COVID-19 vaccine. Multivariable logistic regression was performed using the Forward Likelihood Ratio (Forward LR) method.

Bold values indicate statistically significant associations (*p* < 0.05).

In the analysis of COVID-19-related hospitalization, univariate logistic regression identified older age, dyspnea, colchicine use, disease duration, spondyloarthritis, CoronaVac vaccination, chest X-ray/computed tomography involvement, and radiological findings as significant factors. Multivariable logistic regression demonstrated that older age (OR 1.056, 95% CI 1.022–1.092, *p* = 0.001), dyspnea (OR 2.813, 95% CI 1.194–6.629, *p* = 0.018), chest X-ray/computed tomography involvement (OR 4.252, 95% CI 1.432–12.62, *p* = 0.009), and radiological findings (OR 5.246, 95% CI 2.114–13.02, *p* < 0.001) were independent predictors of hospitalization (Table 5).

**Table 5:** Univariate and multivariate logistic regression analysis of factors associated with COVID-19-related hospitalization.

	Univariate			Multivariate		
	OR	95% CI	*p*	OR	95% CI	*p*
Age	1.055	1.026–1.085	***0.000***	1.056	1.022–1.092	***0.001***
Dyspnea	6.658	3.267–13.567	***0.000***	2.813	1.194–6.629	***0.018***
Colchicine use	0.344	0.118–0.997	***0.049***			
Disease duration (years)	1.036	1.000–1.072	***0.047***			
FMF	0.157	0.021–1.176	0.072			
SpA	2.990	1.016–8.801	***0.047***			
BNT162b2 vaccination	0.670	0.337–1.330	0.252			
CoronaVac vaccination	1.995	1.015–3.920	***0.045***			
Combined vaccination	1.517	0.591–3.890	0.386			
Chest X-ray/CT	14.562	5.903–35.922	***0.000***	4.252	1.432–12.62	***0.009***
Radiological findings	16.535	7.824–34.945	***0.000***	5.246	2.114–13.02	***0.000***

OR, odds ratio; CI, confidence interval; FMF, familial Mediterranean fever; SpA, spondyloarthritis; CT, computed tomography; BNT162b2, Pfizer–BioNTech COVID-19 vaccine. Multivariable logistic regression was performed using the Forward Likelihood Ratio (Forward LR) method.

Bold values indicate statistically significant associations (*p* < 0.05).

## 4 Discussion

In this retrospective observational cohort study, we evaluated the clinical characteristics and outcomes of COVID-19 among patients with IRDs. The principal findings of our study were that older age, dyspnea, and pulmonary radiologic involvement were independently associated with COVID-19-related hospitalization after multivariable adjustment. Pulmonary radiologic involvement was not only more frequent among hospitalized patients but also remained independently associated with hospitalization after multivariable adjustment. This finding highlights the close relationship between pulmonary disease burden and severe COVID-19 outcomes and is consistent with previous studies demonstrating that radiographic evidence of pneumonia is a major determinant of disease severity and healthcare utilization.

Although longer disease duration, FMF, colchicine use, and BNT162b2 vaccination were associated with hospitalization in univariate analyses, these associations were not retained after multivariable adjustment and should therefore be interpreted cautiously. SpA was associated with a higher frequency of COVID-19 infection in the univariate analysis, but this association was not confirmed in the adjusted model.

Previous studies demonstrated that patients with rheumatic diseases may experience heterogeneous COVID-19 outcomes depending on demographic characteristics, comorbidities, and treatment exposure. Gianfrancesco et al. reported that advanced age and comorbid conditions were strongly associated with hospitalization in patients with rheumatic diseases participating in the COVID-19 Global Rheumatology Alliance registry (2). Similarly, Strangfeld et al. identified older age and comorbidities as major predictors of COVID-19-related mortality among patients with rheumatic diseases (4). Consistent with these reports, hospitalized patients in our cohort were significantly older and also had a higher prevalence of chronic lung disease in the univariate comparison. However, pulmonary comorbidity did not remain independently associated with hospitalization after multivariable adjustment. Pulmonary comorbidity has consistently been recognized as an important determinant of adverse outcomes in respiratory infections. Beyond COVID-19, recent evidence from patients with community-acquired pneumonia demonstrated that clinical and laboratory factors associated with pulmonary disease severity are closely related to prognosis and adverse clinical outcomes (18). These findings further support the concept that pre-existing pulmonary involvement may contribute to worse clinical outcomes in patients with inflammatory rheumatic diseases who develop respiratory infections, including COVID-19. Dyspnea also emerged as an independent predictor of COVID-19-related hospitalization in the multivariable analysis. This finding is clinically plausible, as respiratory symptoms frequently reflect more advanced pulmonary involvement and have consistently been associated with severe COVID-19 outcomes in previous studies. Therefore, the presence of dyspnea at the time of infection may help identify patients with inflammatory rheumatic diseases who are at increased risk for hospitalization. Although disease duration was associated with COVID-19-related hospitalization in the univariate analysis, this association was not retained after multivariable adjustment. Nevertheless, hospitalized patients had a significantly longer duration of rheumatic disease than non-hospitalized patients. However, this finding should be interpreted with caution, as disease duration does not necessarily reflect cumulative inflammatory burden or disease severity in a heterogeneous cohort of rheumatic diseases. Patients with longstanding rheumatic disease may have stable, well-controlled disease, whereas others with a shorter disease duration may have active disease requiring intensive treatment. Therefore, disease activity would likely provide a more informative measure than disease duration alone. Since standardized disease activity indices were not consistently available for all patients in the present study, the potential influence of disease activity on COVID-19 outcomes could not be evaluated. Future studies incorporating validated disease activity measures are warranted to better clarify this relationship.

ICU admission remained relatively low in the cohort despite the substantial number of COVID-19-positive patients. The relatively low ICU admission rate observed in our cohort may also reflect improved disease monitoring and early vaccination strategies in patients with IRDs.

Although BMI was slightly higher among hospitalized patients, the difference did not reach statistical significance. This borderline finding may reflect the limited number of hospitalized patients and consequently reduced statistical power. Obesity has consistently been identified as a risk factor for severe COVID-19 in the general population (19, 20). However, studies in patients with inflammatory rheumatic diseases have reported inconsistent results, suggesting that age, comorbidities, and underlying disease characteristics may have a greater influence on clinical outcomes (9, 21). Likewise, smoking status was not associated with hospitalization in our cohort. Previous studies have reported conflicting findings regarding the impact of smoking on COVID-19 severity (22, 23), and our results do not support an independent association in this heterogeneous IRD population.

Our study demonstrated a higher frequency of COVID-19 infection among patients with SpA in the univariate analysis. However, this association was not retained after multivariable adjustment, suggesting that differences in demographic or clinical characteristics rather than disease subtype itself may have contributed to the observed finding. Previous studies have suggested that patients with SpA may exhibit distinct clinical characteristics during COVID-19 infection (8). Differences in age distribution, mobility, biologic treatment exposure, and immune pathways may also contribute to these observations. Accordingly, the observed association between SpA and COVID-19 infection appears more likely to reflect differences in patient characteristics than an independent effect of disease subtype.

Another notable finding was the lower frequency of methotrexate use among COVID-19-positive patients in the univariate analysis. However, this association was not retained after multivariable adjustment, suggesting that it may have been influenced by differences in underlying patient characteristics rather than an independent effect of methotrexate itself. Accordingly, our findings do not support either a protective or harmful independent effect of methotrexate on COVID-19 susceptibility. Previous studies evaluating the effect of conventional DMARDs on COVID-19 outcomes have produced conflicting results. Sparks et al. reported that biologic and targeted synthetic DMARDs were not consistently associated with severe COVID-19 outcomes in rheumatoid arthritis patients (6). Likewise, Conway et al. demonstrated that conventional DMARDs generally did not increase severe COVID-19 risk independently (9). In our study, biologic therapies were not associated with either COVID-19 infection or hospitalization. These findings are consistent with previous reports and suggest that adequately controlled immunomodulatory treatment was not associated with worse COVID-19 outcomes in our cohort. Interestingly, prednisone doses were lower among COVID-19-positive patients in our cohort. One possible explanation is that patients receiving higher corticosteroid doses may have adopted more cautious shielding behaviors during the pandemic. However, this observation may also have been influenced by differences in disease characteristics and other unmeasured confounding factors. Therefore, this finding should not be interpreted as evidence of a protective effect of corticosteroids against SARS-CoV-2 infection.

One of the notable observations in the univariate analyses was the lower hospitalization rate among FMF patients and colchicine users. FMF is characterized by excessive inflammasome activation and dysregulated innate immune responses. Colchicine inhibits neutrophil activation and inflammasome-mediated cytokine release, mechanisms implicated in severe COVID-19-associated hyperinflammation (10). During the pandemic, colchicine emerged as a potential therapeutic option because of these anti-inflammatory properties. Reyes et al. emphasized the theoretical and clinical rationale for colchicine use in COVID-19 (10). In addition, Scarsi et al. reported improved survival among hospitalized COVID-19 patients receiving colchicine therapy (12). Our findings demonstrated an association between colchicine use and lower hospitalization rates; however, because of the observational design, the small number of hospitalized patients, and potential residual confounding, this finding should be considered hypothesis-generating rather than evidence of an independent protective effect. Consistent with this interpretation, neither FMF nor colchicine use remained independently associated with hospitalization after multivariable adjustment. Accordingly, our findings do not support initiating colchicine solely for the prevention of severe COVID-19 in patients with inflammatory rheumatic diseases. Instead, colchicine should continue to be prescribed according to its established rheumatologic indications until further evidence from adequately adjusted prospective studies becomes available. In particular, confounding by indication cannot be excluded, as colchicine use primarily reflected the underlying diagnosis of FMF and may therefore be associated with patient characteristics that independently influenced COVID-19 outcomes.

Vaccination status was another major determinant of COVID-19 outcomes in our cohort. Patients vaccinated with BNT162b2 had lower hospitalization rates in the univariate analysis. However, vaccine type was not independently associated with hospitalization after multivariable adjustment, suggesting that temporal factors, patient characteristics, and other unmeasured confounders may have contributed to the observed association. Previous studies demonstrated that mRNA vaccines induce stronger humoral immune responses compared with inactivated vaccines in patients with autoimmune IRDs. Furer et al. showed satisfactory immunogenicity and safety of the BNT162b2 vaccine in patients with autoimmune IRDs (13). Similarly, Medeiros-Ribeiro et al. demonstrated acceptable but relatively lower immunogenicity rates with the CoronaVac vaccine in autoimmune rheumatic disease populations (24). Our findings are consistent with previous reports; however, the observed association should be interpreted cautiously because temporal factors and residual confounding may have influenced the results. From a clinical perspective, our findings support current recommendations emphasizing SARS-CoV-2 vaccination in patients with inflammatory rheumatic diseases. Although our observational data do not establish superiority of one vaccine over another, they reinforce the importance of maintaining high vaccination coverage in this vulnerable population and ensuring timely booster vaccination according to current national and international recommendations.

These vaccination-related findings should be interpreted with caution. Vaccine availability, national vaccination policies, and the predominant SARS-CoV-2 variants changed considerably throughout the study period. Patients infected during the early phase of the pandemic had limited or no opportunity to receive vaccination. In contrast, those infected later were more likely to have been vaccinated and exposed to different circulating variants. In addition, the interval between vaccination and SARS-CoV-2 infection was not evaluated. Therefore, temporal factors may have influenced the observed associations between vaccination status and COVID-19 outcomes. Furthermore, the extended recruitment period encompassed multiple pandemic waves characterized by different predominant SARS-CoV-2 variants. Viral variants differed in transmissibility, virulence, and immune escape characteristics. Therefore, they may also have influenced the observed clinical outcomes and should be considered when interpreting our findings.

The Turkish Society for Rheumatology COVID-19 Registry conducted by Esatoglu et al. provided important nationwide data regarding COVID-19 among Turkish patients with IRDs (15). Similar to our findings, they identified older age and comorbid conditions as important determinants of severe disease outcomes. Our study additionally observed lower hospitalization rates among patients with FMF, those receiving colchicine, and those vaccinated with BNT162b2 in the univariate analyses. However, these associations were not retained after multivariable adjustment and therefore require cautious interpretation. These observations should be considered hypothesis-generating and require confirmation in larger studies.

This study has several limitations. First, it was conducted at a single center, which may limit the generalizability of our findings to other populations and healthcare settings. Therefore, large multicenter studies with appropriate adjustment for potential confounding factors are needed to validate our findings. Second, standardized disease activity indices and laboratory inflammatory markers were not uniformly available for all patients and therefore could not be incorporated into the analyses. Consequently, we were unable to evaluate the potential influence of disease activity on COVID-19 susceptibility and hospitalization, which may represent an important confounding factor. As a result, some of the observed associations between rheumatic disease characteristics, treatments, and COVID-19 outcomes may have been influenced by differences in underlying disease activity rather than the variables examined. Therefore, these limitations should be considered when interpreting our findings. Third, systematic SARS-CoV-2 screening was not performed throughout the study period. Therefore, asymptomatic or undiagnosed infections may have been missed, potentially resulting in misclassification of some patients in the COVID-19-negative group. In addition, COVID-19 diagnosis was based on different diagnostic approaches, including PCR testing, compatible thoracic imaging findings, and clinical diagnosis according to national guidelines. Although these approaches reflected real-world clinical practice during different phases of the pandemic, this heterogeneity may have resulted in misclassification bias and should be considered when interpreting the findings. Furthermore, temporal factors, including changes in SARS-CoV-2 variants, vaccine availability, and the interval between vaccination and infection, were not evaluated and may have influenced the observed vaccination-related findings. Finally, because of the observational design, causal relationships between treatments and COVID-19 outcomes cannot be established. Residual confounding may still have influenced the observed associations despite standardized data collection. Furthermore, the relatively small number of hospitalized patients may have limited the statistical power of the multivariable analyses and reduced the ability to detect independent associations for some clinical variables. Therefore, the adjusted estimates should be interpreted cautiously and confirmed in larger cohorts. In addition, several variables that were significant in the univariate analyses were no longer associated with hospitalization after multivariable adjustment, suggesting that residual confounding and interrelationships among clinical variables should be considered when interpreting the observed associations.

## 5 Conclusion

In conclusion, older age, dyspnea, and pulmonary radiologic involvement were independently associated with COVID-19-related hospitalization in patients with IRDs. Lower hospitalization rates were observed among patients with FMF, those receiving colchicine, and those vaccinated with BNT162b2 in univariate analyses; however, these associations were not retained after multivariable adjustment and should therefore be interpreted cautiously.

## Data Availability

The raw data supporting the conclusions of this article will be made available by the authors, without undue reservation.
